# Cause and preventability of in-hospital mortality after PCI: A statewide root-cause analysis of 1,163 deaths

**DOI:** 10.1371/journal.pone.0297596

**Published:** 2024-03-27

**Authors:** Francesco Moroni, Milan Seth, Hameem U. Changezi, Milind Karve, Dilip S. Arora, Manoj Sharma, Elizabeth Pielsticker, Aaron D. Berman, Daniel Lee, M. Imran Qureshi, Lorenzo Azzalini, Devraj Sukul, Hitinder S. Gurm

**Affiliations:** 1 Division of Cardiology, Berne Cardiovascular Research Center and Heart and Vascular Center, School of Medicine, University of Virginia, Charlottesville, Virginia, United States of America; 2 Deparment of Medicine, Università Milano-Bicocca, Milan, Italy; 3 Department of Internal Medicine, Division of Cardiovascular Medicine, University of Michigan, Ann Arbor, Michigan, United States of America; 4 McLaren Flint Cardiology, Flint, Michigan, United States of America; 5 Division of Cardiology, Sparrow Health, Lansing, Michigan, United States of America; 6 Corewell Health South Lakeland, St Joseph, Michigan, United States of America; 7 Division of Cardiology, Covenant HealthCare, Saginaw, Michigan, United States of America; 8 Heart and Vascular Service Line, Henry Ford-Jackson, Jackson, Michigan, United States of America; 9 Division of Cardiology, Corewell Health William Beaumont University Hospital, Royal Oak, Michigan, United States of America; 10 McLaren Bay Heart and Vascular, Bay City, Michigan, United States of America; 11 Division of Cardiology, DMC Sinai Grace Hospital, Detroit, Michigan, United States of America; 12 Department of Medicine, Division of Cardiology, University of Washington, Seattle, Washington, United States of America; Saud Al-Babtain Cardiac Centre, SAUDI ARABIA

## Abstract

**Background:**

Mortality is the most devastating complication of percutaneous coronary interventions (PCI). Identifying the most common causes and mechanisms of death after PCI in contemporary practice is an important step in further reducing periprocedural mortality.

**Objectives:**

To systematically analyze the cause and circumstances of in-hospital mortality in a large, multi-center, statewide cohort.

**Methods:**

In-hospital deaths after PCI occurring at 39 hospitals included in the Blue Cross Blue Shield of Michigan Cardiovascular Consortium (BMC2) between 2012 and 2014 were retrospectively reviewed using validated methods. A priori PCI-related mortality risk was estimated using the validated BMC2 model.

**Results:**

A total of 1,163 deaths after PCI were included in the study. Mean age was 71±13 years, and 507 (44%) were women. Left ventricular failure was the most common cause of death (52% of cases). The circumstance of death was most commonly related to prior acute cardiovascular condition (61% of cases). Procedural complications were considered contributing to mortality in 235 (20%) cases. Death was rated as not preventable or slightly preventable in 1,045 (89.9%) cases. The majority of the deaths occurred in intermediate or high-risk patients, but 328 (28.2%) deaths occurred in low-risk patients (<5% predicted risk of mortality). PCI was considered rarely appropriate in 30% of preventable deaths.

**Conclusions:**

In-hospital mortality after PCI is rare, and primarily related to pre-existing critical acute cardiovascular condition. However, approximately 10% of deaths were preventable. Further research is needed to characterize preventable deaths, in order to develop strategies to improve procedural safety.

## Introduction

Percutaneous Coronary Intervention (PCI) is one of the most common procedures performed in the United States and worldwide [1]. Although uncommon, periprocedural mortality is the most serious complication of the procedure. Early experience with PCI in the 1990s suggested that most patients who died after the intervention had experienced a procedural complication [2]. This observation supported the use of periprocedural mortality as a surrogate marker for procedural quality in PCI. Several states have thus implemented public reporting of clinical outcomes after PCI. While advocates suggest that public reporting increases transparency and may improve outcomes in cardiovascular care, others suggest that it may foster unwanted consequences such as the adoption of risk-averse practices which may ultimately prevent patients from receiving procedures that are indicated and potentially lifesaving [3,4].

To add to the controversy, more recent data using standardized, validated approaches to root-cause analysis in the setting of contemporary practice, have shown that periprocedural mortality after PCI is rarely the direct result of a procedural complication, whereas, in most instances, it is due to the increased acuity and comorbid conditions of patients treated in the catheterization laboratory [5,6]. However, a small number of deaths were deemed preventable.

The aim of the present work was to systematically review the causes, circumstances and preventability of death after PCI in a large, state-wide cohort using a validated approach to (1) assess the risks associated with the procedure itself and (2) identify the most common causes of death, with the overarching goal of promoting further research aimed at improving the overall safety of PCI.

## Methods

### Study population

The original study population included all patients undergoing PCI, between January 2012 and December 2014, at 39 hospitals participating in the Blue Cross, Blue Shield of Michigan Cardiovascular Consortium (BMC2) Registry who participated in the mortality review process. Cases with periprocedural mortality (i.e., mortality during/after PCI during the index admission) were the focus of the present analysis. The details of BMC2 database were previously reported [7]. Briefly, the BMC2 is a HIPAA-compliant multi-center, statewide database collecting anonymized data regarding PCI procedural details and outcomes at all non-federal hospitals in the state of Michigan. Data is collected by on-site registered nurse coordinators, and includes demographic and clinical characteristics, procedural details, and in-hospital outcomes of patients undergoing PCI procedures. Data quality and the inclusion of consecutive procedures is ensured by ad hoc queries, random chart reviews, detailed site audits by an experienced nurse auditor, and a series of diagnostic routines included in the database. The University of Michigan IRB has waived the need for prior IRB approval for studies performed using BMC2 data since no patient identifiers are collected [8].

### Data abstraction, cause, circumstance and preventability of death adjudication

Each mortality event was independently reviewed locally by either committee of interventional cardiologists or an operator other than the one who performed the original procedure using standardized forms provided by the BMC2 coordinating site. Anonymized case review data were subsequently forwarded to a central database for subsequent analysis purpose. Each review encompassed 4 major evaluations for each case: appropriateness of the indication to perform PCI, cause of death, circumstance of death, and preventability of death. The appropriateness of PCI indication was evaluated according to intersocietal Appropriate Use Criteria for Coronary Revascularization that were available at that time, as well as consideration of additional clinical data that may be relevant in categorizing appropriateness [9]. Briefly, the indication to PCI was categorized as “appropriate”, “may be appropriate” or “rarely appropriate” according to guideline indication.

Cause of death was determined to be ventricular failure, arrhythmic, neurological, respiratory, infectious, hemorrhagic/vascular, or “other” for each patient, in accordance to previously validated methods [5]. The cause of death was defined as the precipitating factor for the patient’s demise, independent of the presenting problem on admission. In case the reviewing committee felt unable to attribute death to a single cause, more than one cause could be listed.

Death was ascribed to one or more of the following circumstances [5]:

1. Procedural complication: new disease state or acute worsening of a pre-existing disease state directly attributable to the procedure (e.g., coronary perforation).
2. Pre-existing acute cardiovascular cause: new cardiovascular diagnosis within the 14 days before PCI which was not worsened by the procedure (e.g., acute myocardial infarction).
3. Pre-existing chronic cardiovascular cause: pre-existing cardiovascular condition diagnosed >14 days before PCI which was not acutely worsened by the procedure (e.g., chronic heart failure).
4. Pre-existing, non-cardiovascular cause: pre-existing non-cardiovascular condition, either acute or chronic (e.g., cancer).
5. Post-procedural complication: event occurring during the hospital stay, after the procedure and not related to it (e.g., ventilator-associated pneumonia).

**Table 1** summarizes causes and circumstances of death. The preventability of death was determined by each reviewer based on a thorough review of each patient’s medical record. Preventability was graded using a previously validated 6-point Likert scale [5]: 0 was defined as an unpreventable death; 1, slightly preventable; 2, moderately preventable; 3, mostly preventable; 4, strongly preventable; and 5, entirely or certainly preventable. We defined the concept of preventability as follows: “including or subsequent to the initial decision to perform cardiac catheterization, the managing physician could have pursued a different treatment plan, thereby impacting mortality.” This was included in the instructions to reviewers with the data collection form and provided examples as previously described by Valle and colleagues [5]. An example of entirely preventable deaths might be air embolization triggering hemodynamic collapse and death while an unpreventable death might include a patient presenting with out of hospital cardiac arrest, undergoing successful PCI and subsequently dying from anoxic brain injury.

**Table 1:** Causes and circumstances of death, as adjudicated by reviewers.

**Causes of death**	
Ventricular failure • Left • Right	
Arrhythmia	
Neurological cause	
Respiratory failure	
Infection	
Hemorrhagic/Vascular	
Other	
**Circumstance of death**	
Procedural complication	New disease state or acute worsening of a pre-existing disease state directly attributable to the procedure (e.g., coronary perforation).
Pre-existing acute cardiovascular	New cardiovascular diagnosis within the 14 days before PCI which was not worsened by the procedure (e.g., acute myocardial infarction).
Pre-existing, chronic cardiovascular	Pre-existing cardiovascular condition diagnosed >14 days before PCI which was not acutely worsened by the procedure (e.g., chronic heart failure).
Pre-existing, noncardiovascular	Pre-existing non-cardiovascular condition, either acute or chronic (e.g., cancer).
Post-procedural complication	Event occurring during the hospital stay, after the procedure and not related to it (e.g., ventilator-associated pneumonia).

In addition to the aforementioned evaluation, each patient’s “a priori” risk of PCI-associated mortality was estimated based on pre-procedural clinical characteristics using the validated BMC2 mortality prediction model [10]. A procedure was considered to be low risk if the predicted risk of death was <5%.

### Statistical analysis

Continuous variables are reported as mean ± standard deviation, and categorical variables as number and percentage. The independent-samples Student’s t-test was used to compare continuous variables with normal distribution, and the Wilcoxon signed rank test was used to compare continuous variables with a non-normal distribution. Chi-square test was used to compare differences between categorical variables. To assess differences in baseline covariate distributions, the Absolute Standardized Difference (ASD) statistic was reported in addition to the p-value for a statistical comparison [11]. The ASD provides a measure univariate imbalance that is robust to the impact of large sample size seen in statistical testing, where very small and likely insignificant differences can reach statistical significance (ie p< 0.05) in large samples. An ASD of ≥ 10% was used to identify variables with substantial imbalance between groups. A 2-tailed p-value <0.05 was considered statistically significant. All analyses were performed with R version 4.1.0 (R Core Team, Vienna, Austria).

## Results

Among 91,308 unique PCIs performed during the study period at the 39 centers contributing to the present analysis, 1,386 deaths occurred, resulting in an overall in-hospital mortality after PCI of 1.5%. Of these deaths, 1,163 (83.9%) were independently reviewed. Death occurred within 19 days from the procedure in 95% of cases, and median length of stay after the procedure was 2 days. Average age was 71±13 years, 507 (44%) were women and 85% self-identified as white. Compared with patients discharged alive, patient who died in the hospital were significantly older (71±13 vs 65±12 years of age, p<0.001) and were more likely to have diabetes (45% vs 39%, p<0.001), chronic lung disease (23% vs 19%, p = 0.002), chronic kidney disease (mean estimated glomerular filtration rate [eGFR] 52 vs 70 ml/min, p<0.001) and had a history of congestive heart failure (27% vs 17%, p<0.001) or peripheral artery disease (22% vs 16%, p<0.001). Notably, patient who died tended to have more impaired left ventricular function (left ventricular ejection fraction 37±17% vs 52±13%, p<0.001). All baseline clinical characteristics of the patient population are summarized in **Table 2**.

**Table 2:** Baseline clinical characteristics.

	Discharged alive	Deceased (with death review)	P-value	ASD (%)
N	89,922	1,163		
Females	29841 (33.2)	507 (43.6)	<0.001	21.50%
Age—mean (SD)	65.11 (11.96)	71.13 (12.85)	<0.001	48.50%
Race/ethnicity:				
White (%)	76979 (85.6)	990 (85.1)	0.672	1.40%
Black (%)	10257 (11.4)	147 (12.6)	0.205	3.80%
Asian (%)	932 (1.0)	13 (1.1)	0.899	0.80%
Hispanic origin (%)	1173 (1.3)	23 (2.0)	0.064	5.20%
BMI—mean (SD)	30.61 (7.30)	29.34 (10.38)	<0.001	14.10%
Current smoker (%)	26220 (29.2)	301 (25.9)	0.016	7.30%
Diabetes (%)	34891 (38.8)	524 (45.1)	<0.001	12.70%
Dyslipidemia (%)	73889 (82.2)	785 (67.6)	<0.001	34.30%
Chronic Lung Disease (%)	17369 (19.3)	267 (23.0)	0.002	8.90%
eGFR—mean (SD)	69.80 (24.93)	52.65 (26.50)	<0.001	66.60%
Currently on dialysis (%)	2180 (2.4)	66 (5.7)	<0.001	16.60%
Prior CHF (%)	14936 (16.6)	312 (26.8)	<0.001	25.00%
Prior MI (%)	31807 (35.4)	397 (34.2)	0.408	2.50%
Prior PAD (%)	14559 (16.2)	260 (22.4)	<0.001	15.70%
Prior PCI (%)	41519 (46.2)	370 (31.8)	<0.001	29.80%
Prior CABG (%)	16827 (18.7)	163 (14.0)	<0.001	12.70%
LVEF—mean (SD)	52.01 (12.79)	37.20 (16.94)	<0.001	98.70%

BMI, body mass index; CABG, coronary artery bypass graft; CHF, congestive heart failure; eGFR, estimated glomerular filtration rate; LVEF, left ventricular ejection fraction; MI, myocardial infarction; PAD, peripheral artery disease; PCI, percutaneous coronary intervention; SD, standard deviation.

Patient who died in hospital were more likely to present with acute coronary syndrome: 28% presented with non-ST-elevation myocardial infarction, while 58% presented with ST-elevation myocardial infarction. As expected, the proportion of patients presenting with myocardial infarction was higher among patients who died compared with patients who were discharged alive (86% vs 37%, p<0.001). The a priori predicted risk of mortality was significantly higher among patients who died compared with those who did not (22.9±21.0% vs 1.2±4.8%, p<0.001). PCI indication and a priori risk are summarized in **Table 3**.

**Table 3:** Clinical presentation and procedural characteristics.

	Discharged alive	Deceased (with death review)	P-value	ASD (%)
N	89,922	1,163		
Predicted risk of death (percent)—mean (SD)	1.18 (4.32)	22.99 (21.04)	<0.001	143.60%
CAD Presentation (%)			<0.001	128.60%
No symptom, no angina	3719 (4.1)	29 (2.5)		
Symptom unlikely to be ischemic	1986 (2.2)	13 (1.1)		
Stable angina	9141 (10.2)	16 (1.4)		
Unstable angina	41569 (46.2)	109 (9.4)		
NSTEMI	20431 (22.7)	320 (27.5)		
STEMI or equivalent	13061 (14.5)	676 (58.1)		
Cardiogenic shock on presentation	1559 (1.7)	556 (47.8)	<0.001	1.262
Radial access	22407 (24.9)	96 (8.3)	<0.001	0.46
Left Main PCI (%)	2419 (2.7)	126 (10.8)	<0.001	32.90%
Multivessel PCI (%)	10240 (11.4)	224 (19.3)	<0.001	22.00%
Atherectomy device used (%)	991 (1.1)	22 (1.9)	0.016	6.50%
MCS (%)			<0.001	51.10%
MCS in place at start of procedure	79 (0.1)	24 (2.1)		
MCS inserted after PCI has begun	76 (0.1)	59 (5.1)		
MCS inserted during procedure and prior to PCI	521 (0.6)	73 (6.3)		
No MCS	89246 (99.2)	1007 (86.6)		
Aspirin	86793 (96.5)	1042 (89.6)	<0.001	0.275
P2Y12 inhibitor				
Clopidogrel	58915 (65.5)	609 (52.4)	<0.001	0.27
Prasugrel	16548 (18.4)	62 (5.3)	<0.001	0.413
Ticagrelor	11209 (12.5)	119 (10.2)	0.025	0.07
GP IIb/IIIa inhibitor	21534 (23.9)	496 (42.6)	<0.001	0.405
Periprocedural anticoagulation				
Low molecular weight heparin	2974 (3.3)	36 (3.1)	0.75	0.012
Unfractionated Heparin	68435 (76.1)	992 (85.3)	<0.001	0.235
Bivalirudin	40098 (44.6)	346 (29.8)	<0.001	0.311
Direct thrombin inhibitor (other)	228 (0.3)	5 (0.4)	0.373	0.03

ASD = absolute standardized difference; CAD = coronary artery disease; MCS = mechanical circulatory support; NSTEMI = non-ST elevation myocardial infarction; PCI = percutaneous coronary intervention; SD = standard deviation; STEMI = ST elevation myocardial infarction.

### Appropriateness of PCI indication, causes, circumstances and preventability of death

**Fig 1:**
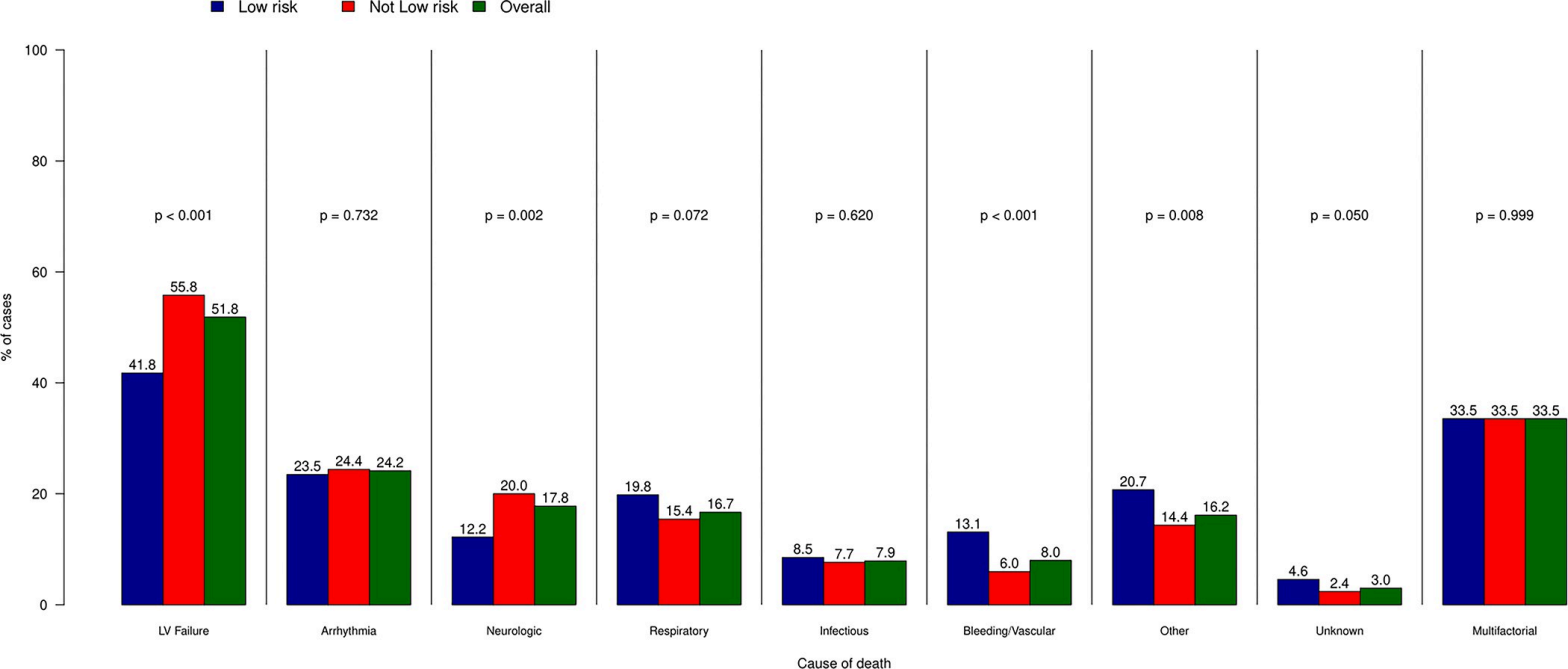
Causes of death in the study population. Each bar represents the percentage of each adjudicated cause of death for the overall population (green) and stratified per a priori risk of death (blue, low risk i.e. <5% of a priori estimated risk of death and red, non-low risk procedures). LV = left ventricular.

PCI indication was considered appropriate, i.e. class I or II according to the appropriate use criteria, in 1,071 (92.1%) of cases. Cause of death was considered multi-factorial in 390 (33.5%) cases. The most commonly observed cause of death was left ventricular failure, occurring in 603 (51.8%) of cases. Other common causes of death were fatal arrhythmias in 281 (24.2%) patients, neurologic complications in 207 (17.8%) and respiratory failure, which occurred in 194 (16.7%) patients (**Fig 1)**.

**Fig 2:**
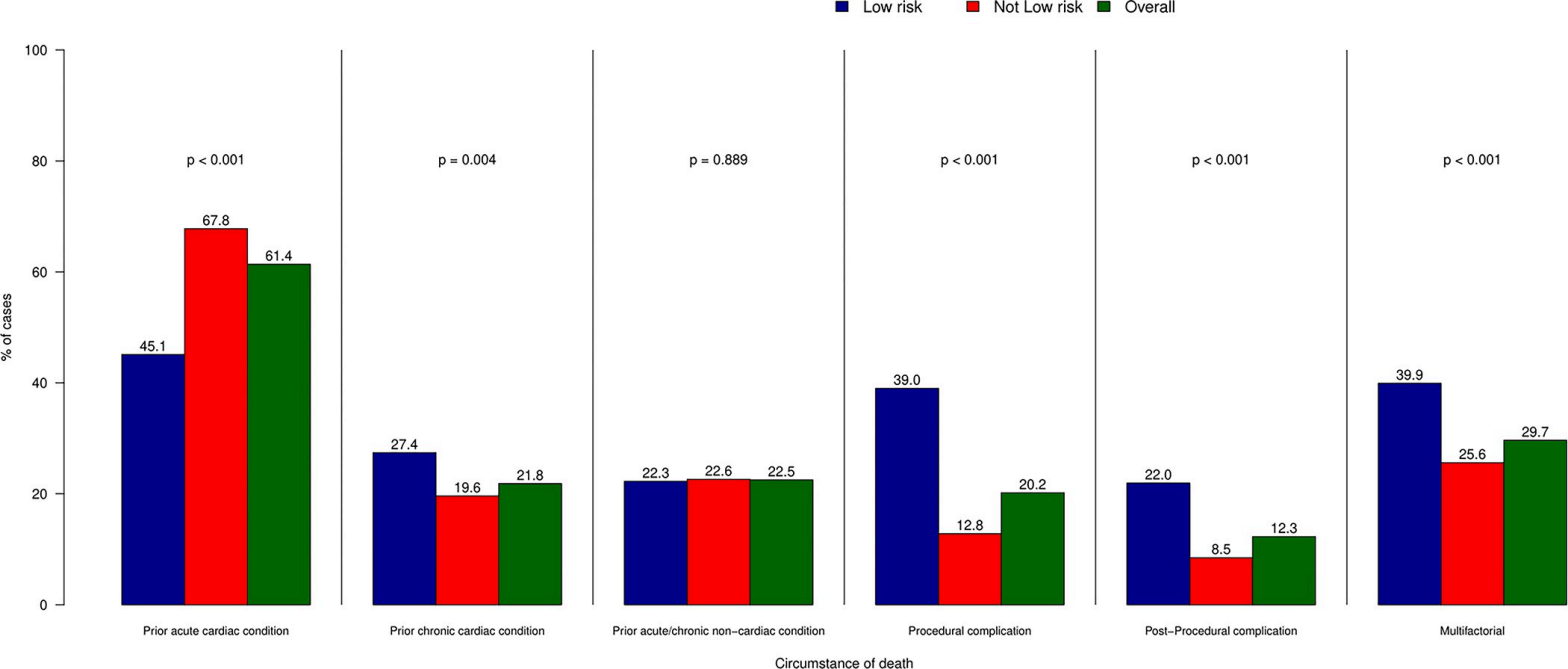
Circumstances of death in the study population. Each bar represents the percentage of each adjudicated circumstance of death for the overall population (green bar) and stratified per a priori risk of death (blue, low risk i.e. <5% of a priori estimated risk of death and red, non-low risk procedures).

Circumstances of death were considered multifactorial in 345 (29.6%) subjects. The most common circumstance of death was a pre-existing acute cardiac condition, which was present in 714 (61.4%) cases. Other common circumstances of death were prior chronic cardiac condition (in 254 [21.8%] subjects) and prior acute or chronic non-cardiac conditions (in 262 [22.5%] individuals). Procedural complications were considered to contribute to the circumstance of death in 235 (20.2%) cases. **Fig 2** shows circumstances of death. Death was rated as unpreventable or only slightly preventable in 1,045 (89.9%) of cases.

### A priori PCI risk and appropriateness, causes, circumstances and preventability of death

According to the validated BMC2 mortality prediction model, 328 (28.2%) deaths occurred after low-risk procedures. Notably, low-risk PCI were more likely to be rated as rarely appropriate (11.0% vs 6.1%, p<0.001). Cause of death in low-risk PCI was less likely to be left ventricular failure compared to non-low risk procedures (41.8% vs 55.8%, p<0.0001). On the other hand, bleeding and vascular complications were more commonly observed (13.1% vs 5.9%, p = 0.0001). Similarly, circumstances of death were more commonly multifactorial (39.9% vs 25.6%, p<0.0001), and encompassed prior acute cardiovascular conditions less frequently than non-low risk procedures (45.1% vs 67.8%, p<0.0001). Procedural complications and post-procedural complications were more common in patients dying after low-risk PCI (39.0% vs 12.8%, p<0.0001 and 21.9% vs 8.5%, p<0.0001, respectively).

In terms of procedural complications, catheter-induced coronary dissection was reported in 5 (0.4%) cases, while coronary perforation in 3 (0.3%) patients. Bleeding events were reported in 58 (4.9%) subjects, and only 1 periprocedural stroke was reported.

Death was rated as largely preventable more frequently in low-risk interventions compared to non-low risk procedures (15.8% vs 5.3%, p<0.0001).

### Preventable deaths after PCI

**Fig 3:**
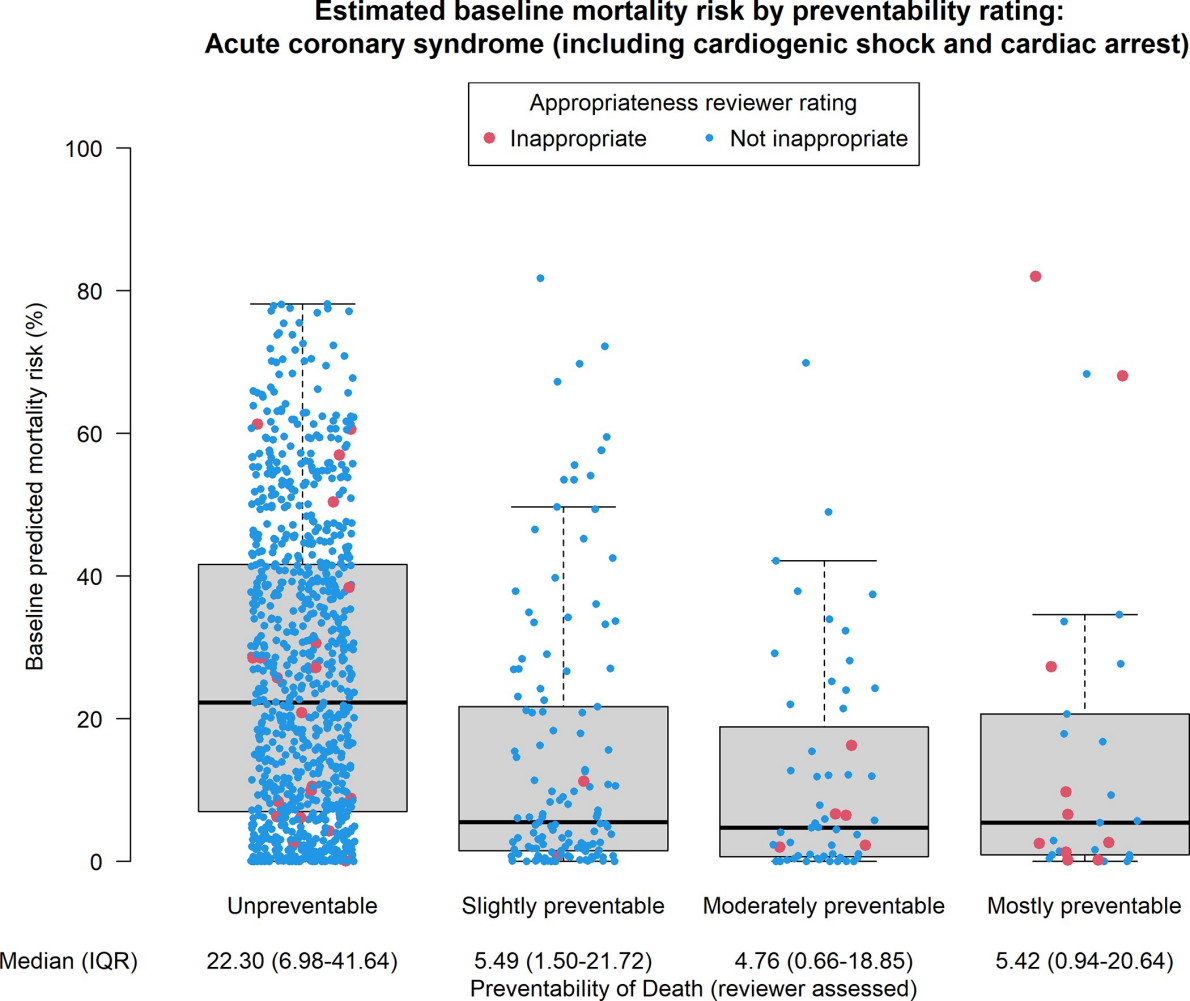
Relation between preventability, appropriateness and a priori estimate of the risk of death. Each dot represent a single case. Adjudicated preventability of death is plotted on the x-axis as an ordinal variable (from unpreventable to mostly preventable). A priori the risk of death using the BMC2 model is plotted on the y-axis. Inappropriate PCI indications are indicated by a red dot, while non-inappropriate cases are in light blue. The underlying box plot shows median (thick line), interquartile range (box) and range (whiskers) of the distribution of estimated risk of mortality. Median and interquartile range are reported as explicit numbers at the bottom of the plot. BMC2, Blue Cross Blue Shield of Michigan; IQR, interquartile range.

Of the 96 deaths after PCI that were considered largely preventable, 62 (64%) were considered moderately preventable, 33 (34%) mostly preventable and only 1 death strongly preventable and attributed to vascular complications related to the intervention. Cause of death was multi-factorial in 32 (33%) of cases. LV failure remained the most common cause of death, occurring in approximately 36 (37%) of cases, followed by arrhythmia in 25 (26%) of cases and bleeding and vascular complications in 22 (23%) of cases. Circumstance of death was most commonly procedural complications (70% of deaths). Notably, coronary perforation was indicated as the primary cause of death in 3 patients, while coronary dissection in 4 patients. Post-procedural complications accounted for 28% of preventable deaths, while non-cardiac causes accounted for 9%. PCI was considered rarely appropriate in 30% of preventable deaths. **Fig 3** shows the relationship between preventability, appropriateness and a priori estimated risk, while **S1 Fig** displays the same relationship in the elective subgroup and **S2 Fig** displays the relationship in the emergent subgroup.

## Discussion

The main results of the present work are: 1) in-hospital death after elective PCI is uncommon, 2) death most commonly occurs in patients who present acutely with myocardial infarction, especially if complicated by shock, and is most commonly secondary to a pre-existing acute cardiovascular condition, 3) in the vast majority of cases, death was considered unpreventable, and 4) deaths in low-risk patients undergoing PCI for rarely appropriate indications were most likely to be preventable. Our findings thus corroborate and extend prior work in two important ways.

Firstly, our findings from a statewide analysis of mortality following PCI confirm work from other contemporary reports. Our findings are in stark juxtaposition to the early PCI experience published in the 1990s, when the majority of deaths were ultimately traceable to procedural complications [2,12]. Indeed, similar to contemporary reports of PCI practice, the majority of deaths were attributed to pre-existing acute conditions [5,6,13]. In the work by Valle et al., approximately 92% of the 85 deaths analyzed were not attributable to any procedural factor but either imputable to a pre-existing acute cardiac condition (most commonly, in approximately 63% of cases) or a post-procedural, noncardiac complication (approximately 16% of cases) [5]. Notably, death was rated as unpreventable or only slightly preventable in 69% of cases [5]. Moroni et al. recently reported similar findings in a cohort of 166 subjects [13]. Notably, in the latter study 93% of subjects had presented with acute myocardial infarction, and 45% were in overt shock at the time of the procedure [13]. Indeed, the majority of deaths were unpreventable or slightly preventable, and secondary to pre-existing acute cardiovascular conditions. [13] Finally, Bricker et al. reported on 1,674 deaths in the 30-day period following PCI [6]. In an experience from the Veteran Affairs Healthcare System, up to 74% of deaths were considered unpreventable, and only one-third were attributable to cardiac causes. Only 8% were traceable to a procedural complication [6]. Our results confirm and expand findings from previous literature. First, our study population was very large, comparable to that of Bricker et al., but encompassed a higher proportion of female subject, and encompassed institutions varying from tertiary referral centers, academic centers and community hospitals contributing to the generalizability of our results.

Second, while uncommon, preventable deaths do occur and a significant proportion (approximately 30%) of preventable deaths occurred in procedures that were deemed to be of low value and could potentially be avoided. In proportion, preventable deaths were more likely to occur in the setting of procedural complications. Notably, however, coronary perforation, which is among the most dreaded PCI complication, was exceedingly rare, as previously reported [14]. These data suggest that systems focusing on optimizing appropriateness of PCI might be an effective strategy to reduce preventable deaths from PCI. In addition, deaths in patients deemed to be of low predicted risk of mortality were more likely to have a higher likelihood of being preventable and a targeted assessment of such procedures might be more effective at optimizing procedural safety than a broader approach of reviewing all deaths at mortality and morbidity conferences that is commonly followed in many hospitals. Along those same lines, risk guided approaches might be also impactful for guiding regional and national focused quality efforts and research studies. Finally, given the infrequent occurrence of preventable death, our data support a more nuanced approach to using mortality for public reporting and ranking institutions on quality [15].

### Limitations

The present work does have some limitations that need to be acknowledged. First, approximately 16% of death cases within the study period were not included in the analysis, due to inability of some centers to develop the appropriate process for case review. Second, case review and preventability of death adjudication were performed locally, which may have introduced some heterogeneity in the classification of deaths. The use of centrally generated, standardized forms as well as centralized training on root-cause analysis and event adjudication may have mitigated the latter. Our data originate from a State with a strong culture of quality improvement and collaboration and may or may not be directly applicable to all types of clinical practice patterns [16]. Finally, some of the data has been collected and generated close to a decade ago, which may not fully reflect contemporary practice and indications for PCI.

## Conclusion

In-hospital mortality after PCI is rare, and mainly secondary to pre-existing critical acute condition, with procedural complications being a contributing factor in one-fifth of cases. However, 10% of deaths were considerate preventable, and was often observed in patients undergoing low risk procedures deemed to be poorly rated on appropriateness. Our data support further research to characterize preventable deaths in order to develop strategies to further enhance procedural safety.

## Supporting information

S1 Fig Estimated baseline mortality risk by preventability: Elective PCI. This plot shows median (thick line), interquartile range (box) and range (whiskers) of the distribution of estimated risk of mortality among the subset of patients undergoing elective percutaneous coronary intervention (PCI). Each dot represents a single case. Adjudicated preventability of death is plotted on the x-axis as an ordinal variable (from unpreventable to mostly preventable). A priori the risk of death using the BMC2 model is plotted on the y-axis. Inappropriate PCI indications are indicated by a red dot, while non-inappropriate cases are in light blue. Median and interquartile range for the a priori risk of death are reported as explicit numbers at the bottom of the plot. BMC2, Blue Cross Blue Shield of Michigan; IQR, interquartile range.
(DOCX)

S2 Fig Estimated baseline mortality risk by preventability rating: Acute coronary syndrome. This plot shows median (thick line), interquartile range (box) and range (whiskers) of the distribution of estimated risk of mortality among the subset of patients undergoing percutaneous coronary intervention (PCI) for an urgent or emergent indication, including acute coronary syndromes, cardiogenic shock and cardiac arrest. Each dot represents a single case. Adjudicated preventability of death is plotted on the x-axis as an ordinal variable (from unpreventable to mostly preventable). A priori the risk of death using the BMC2 model is plotted on the y-axis. Inappropriate PCI indications are indicated by a red dot, while non-inappropriate cases are in light blue. Median and interquartile range for the a priori risk of death are reported as explicit numbers at the bottom of the plot. BMC2, Blue Cross Blue Shield of Michigan; IQR, interquartile range.
(DOCX)

## Abbreviations

PCI: percutaneous coronary intervention
BMC2: Blue Cross, Blue Shield of Michigan Cardiovascular Consortium
HIPAA: Health Insurance Portability and Accountability Act of 1996
IRB: institutional review board
ASD: Absolute Standardized Difference
