# Supplementary figures and images for: Cause and preventability of in-hospital mortality after PCI: A statewide root-cause analysis of 1,163 deaths
Source: PLoS One. 2024 Mar 27;19(3):e0297596. doi: 10.1371/journal.pone.0297596 (PMC10971674; doi:10.1371/journal.pone.0297596)

**S1 Fig. Estimated baseline mortality risk by preventability: Elective PCI**

**
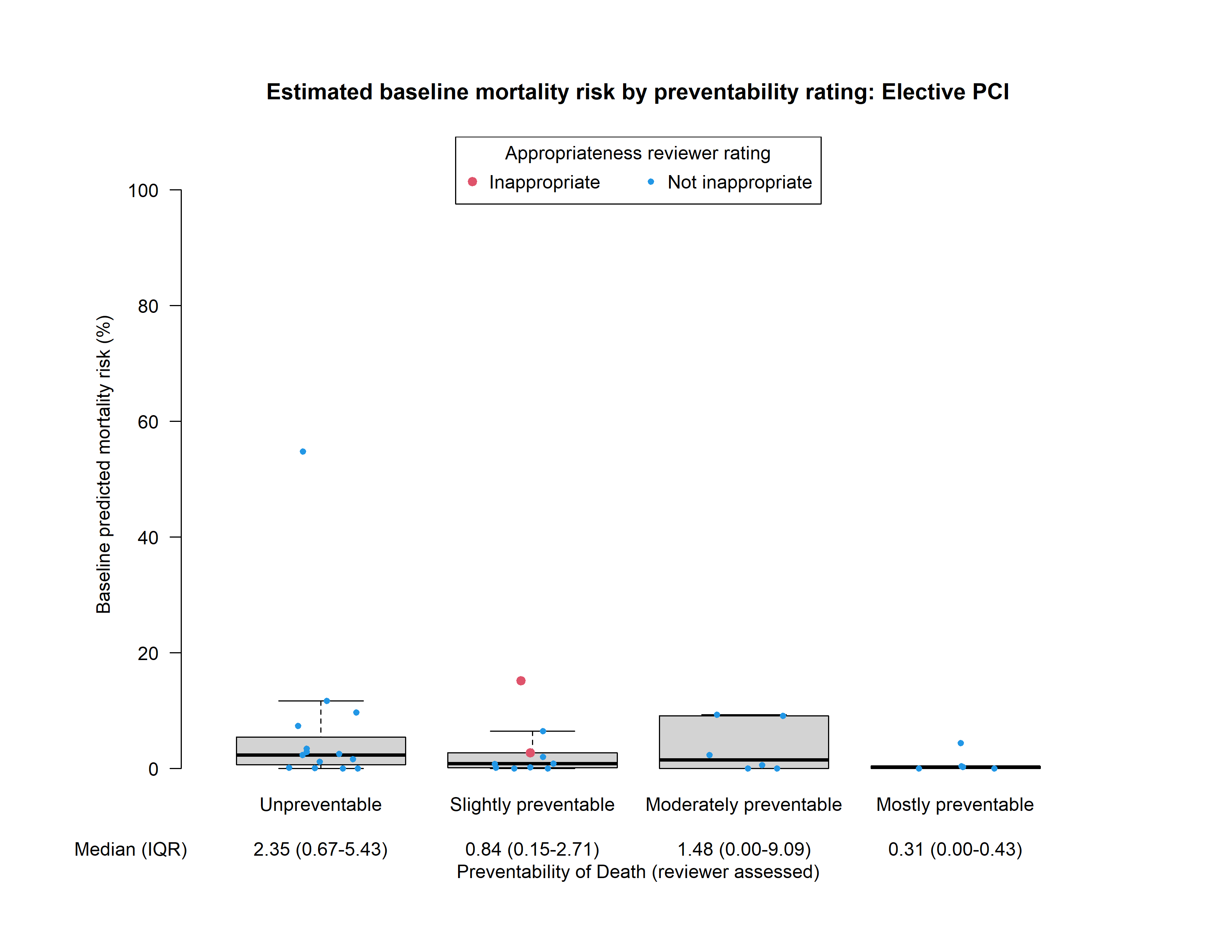
**

Supplement: S1 Fig — This plot shows median (thick line), interquartile range (box) and range (whiskers) of the distribution of estimated risk of mortality among the subset of patients undergoing elective percutaneous coronary intervention (PCI). Each dot represents a single case. Adjudicated preventability of death is plotted on the x-axis as an ordinal variable (from unpreventable to mostly preventable). A priori the risk of death using the BMC2 model is plotted on the y-axis. Inappropriate PCI indications are indicated by a red dot, while non-inappropriate cases are in light blue. Median and interquartile range for the a priori risk of death are reported as explicit numbers at the bottom of the plot. BMC2, Blue Cross Blue Shield of Michigan; IQR, interquartile range. (DOCX) [file pone.0297596.s001.docx]

**S2 Fig. Estimated baseline mortality risk by preventability rating: Acute coronary syndrome**

**
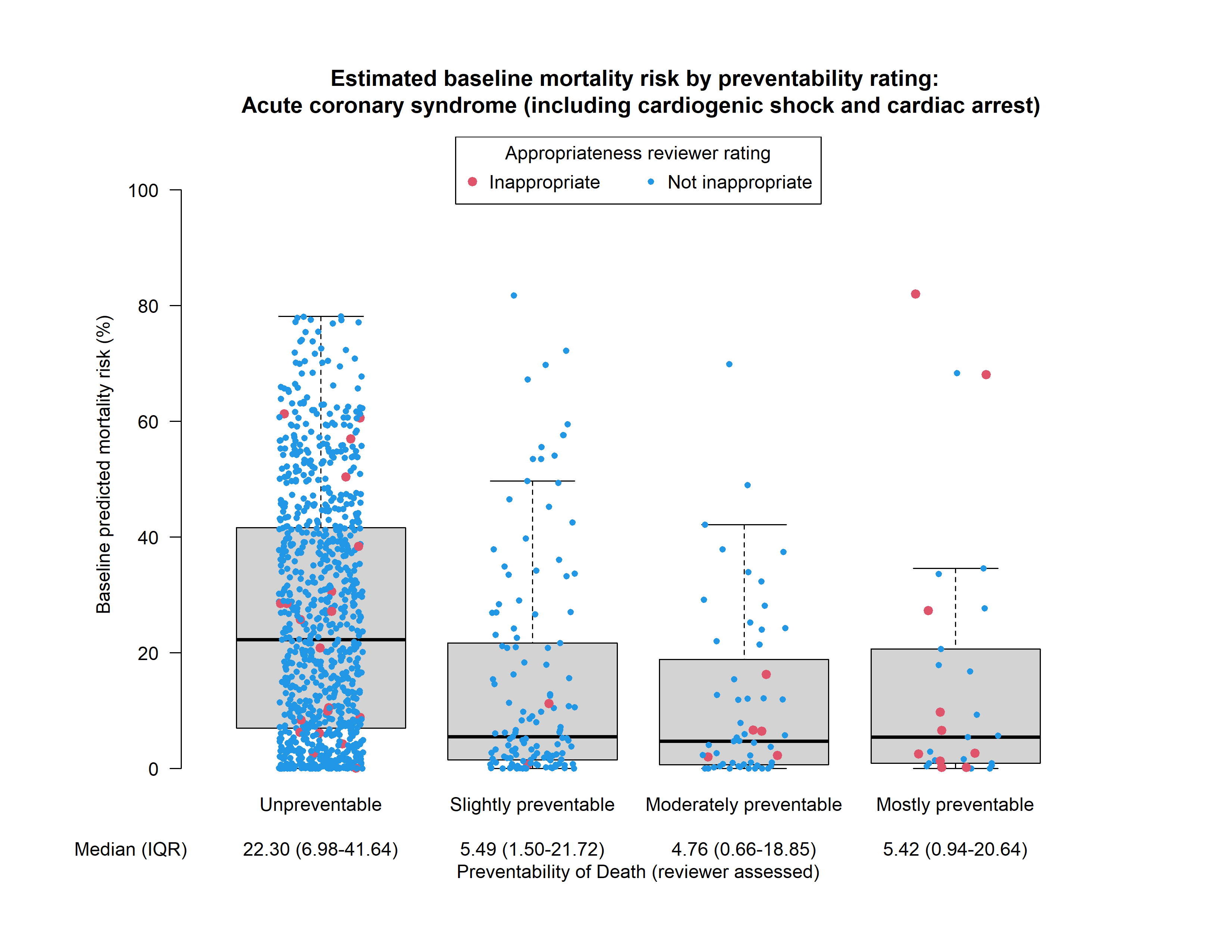
**

Supplement: S2 Fig — This plot shows median (thick line), interquartile range (box) and range (whiskers) of the distribution of estimated risk of mortality among the subset of patients undergoing percutaneous coronary intervention (PCI) for an urgent or emergent indication, including acute coronary syndromes, cardiogenic shock and cardiac arrest. Each dot represents a single case. Adjudicated preventability of death is plotted on the x-axis as an ordinal variable (from unpreventable to mostly preventable). A priori the risk of death using the BMC2 model is plotted on the y-axis. Inappropriate PCI indications are indicated by a red dot, while non-inappropriate cases are in light blue. Median and interquartile range for the a priori risk of death are reported as explicit numbers at the bottom of the plot. BMC2, Blue Cross Blue Shield of Michigan; IQR, interquartile range. (DOCX) [file pone.0297596.s002.docx]
